# Production of poly(3-hydroxybutyrate) by *Halomonas boliviensis* in an air-lift reactor

**DOI:** 10.1186/s40709-015-0031-6

**Published:** 2015-08-03

**Authors:** Paola Rivera-Terceros, Estefanía Tito-Claros, Sonia Torrico, Sergio Carballo, Doan Van-Thuoc, Jorge Quillaguamán

**Affiliations:** Center of Biotechnology, Faculty of Sciences and Technology, San Simon University, Cochabamba, Bolivia; Center of Agroindustrial Technology, Faculty of Sciences and Technology, San Simon University, Cochabamba, Bolivia; Center of Food and Natural Products, Faculty of Sciences and Technology, San Simon University, Cochabamba, Bolivia; Department of Microbiology and Biotechnology, Faculty of Biology, Hanoi National University of Education, 136 XuanThuy, CauGiay, Hanoi, Vietnam

**Keywords:** PHB production, Air-lift bioreactor, Starch hydrolysate, Halophile, *Halomonas boliviensis*

## Abstract

**Background:**

Microbial polyesters, also known as polyhydroxyalkanoates (PHAs), closely resemble physical and mechanical features of petroleum derived plastics. Recombinant *Escherichia coli* strains are being used in industrial production of PHAs in Stirred Tank Bioreactors (STRs). However, use of Air-Lift Reactors (ALRs) has been known to offer numerous technical operating options over STRs, and as such has been successfully implemented in many bioprocesses. *Halomonas boliviensis* is a halophilic bacterium that is known to assimilate various carbohydrates and convert them into a particular type of PHA known as poly(3-hydroxybutyrate) (PHB). Owing to this capability, it has been used to synthesize the polyester using hydrolysates of starch or wheat bran in stirred tank bioreactors.

**Results:**

This research article firstly describes the production of PHB in shake flasks by *H. boliviensis* using different combinations of carbohydrates and partially hydrolyzed starch as carbon sources. The highest PHB yields, between 56 and 61 % (wt.), were achieved when either starch hydrolysate or a mixture of glucose and xylose were used as carbon sources. The starch hydrolysate obtained in this study was then used as carbon source in an ALR. The largest amount of PHB, 41 % (wt.), was attained after 24 hrs of cultivation during which maltose in the hydrolysate was assimilated more rapidly than glucose during active cell growth; however, the rate of assimilation of both the carbohydrates was found to be similar during synthesis of PHB. An incomplete pentose phosphate pathway, which lacks 6-phosphogluconate dehydrogenase, was deduced from the genome sequence of this bacterium and may result in the characteristic assimilation of glucose and maltose by the cells.

**Conclusions:**

This study showed that the production of PHB by *H. boliviensis* using cheap substrates such as starch hydrolysate in a simple production system involving an ALR is feasible. Both maltose and glucose in the hydrolysate induce cell growth and PHB synthesis; most likely the cells balance adequately CoA and NAD(P)H during the assimilation of these carbohydrates. The combination of cheap substrates, simple production systems and the use of non-strict sterile conditions by the halophile *H. boliviensis* are desirable traits for large scale production of PHB, and should lead to a competitive bioprocess.

**Electronic supplementary material:**

The online version of this article (doi:10.1186/s40709-015-0031-6) contains supplementary material, which is available to authorized users.

## Background

Microbial polyesters, also known as polyhydroxyalkanoates (PHAs), are biodegradable plastic-like materials whose physical and mechanical features closely resemble those of petroleum derived plastics such as polyethylene and polystyrene [1]. PHAs are synthesized by microorganisms, as carbon and energy storage compounds, from renewable sources such as carbohydrates and fatty acids [1, 2]; therefore PHAs have found applications in fine chemistry, biofuels, nanotechnology and biomedicine [2, 3]. A carbon source in excess and the depletion of an essential nutrient, such as nitrogen, phosphorus or oxygen, in the medium usually induce the polymer synthesis in individual cells [1]. Large scale production of PHAs has been attained; however, the commercial competitiveness of PHAs has encountered its main drawback in elevated processing costs, which have been related to the polymer yield reached by a microorganism, carbon source employed, production system and polymer purification [1, 2].

Recombinant *Escherichia coli* strains are being used in industrial production of PHAs in which stirred tank bioreactors are fed with glucose or sucrose as the main carbon source for cell growth and PHA production [2]. There are nonetheless other bacterial strains that are considered good candidates for large scale production of PHAs, among them *Cupriavidus necator* (formerly named *Ralstonia eutropha*), *Azohydromonas australica* (formerly known as *Alcaligenes latus*), *Pseudomonas* spp. and *Bacillus* spp. [2, 4]. These microorganisms are known to reach PHA yields of 60-88 % (wt.) in bioreactors operated under a fed-batch mode [1, 2]. Recently, members of the family *Halomonadaceae*, such as *Halomonas boliviensis* [5], *Halomonas* sp. TD01 [6] and *Halomonas* sp. KM-1 [7], have been found to accumulate large amounts of PHAs from various carbon sources. These *Halomonas* species require NaCl in concentrations ranging from 1 to 8 % (w/v) for optimum growth, because they are halophilic microorganisms [8]. High salt concentrations in culture media inhibit the growth of non-halophilic microorganisms, thereby leading to production processes that do not require strict sterile conditions [6, 9]. Consequently, costs involved in the energy consumption intended for the sterilization of bioreactors and tubings should decrease [9]. Hitherto, the highest volumetric productivities achieved by *Halomonas* spp. have been obtained using Stirred Tank Reactors (STRs) [5].

Use of Air-Lift Reactors (ALRs), on the other hand, is known to offer numerous technical options over STRs, and such has been successfully implemented in various bioprocesses [10, 11]. For example, ALRs containing the proliferating microorganisms are pneumatically mixed by air bubbles pumped through the liquid culture media [10], thus providing an alternative to the rotating shaft or impellers used in STRs [10]. Advantages associated with ARLs include low shear stress, simplicity of design and construction, and low energy requirement for mass transfer [10]. ALRs may hence reduce construction and operation costs [10]. ALRs have been used to culture *C. necator* [12], *Burkholderia sacchari* [13] and *A. australica* [14] for poly(3-hydroxybutyrate) (PHB), the most common type of the PHAs, production. *Cupriavidus necator* reached high PHB contents, ~70 % (wt.), when a combination of glucose and sucrose was used as carbon sources and an air flow rate of at least 35 L min^−1^ was supplied to the reactor - PHB accumulation in *C. necator* cells was reduced below such air flow rate [12]. Using a step-wise addition of sucrose in an ALR, *B. sacchari* achieved a high cell density (cell dry weight, CDW, 150 g L^−1^) [13], but a lower PHB yield [42 % (wt.)] than *C. necator* [12]. On the other hand, *A. australica* was able to store up to 73 % (wt.) PHB intracellularly upon achieving a CDW of 10.8 g L^−1^ [14]. These reports have shown the feasibility of the production of PHAs in ALRs.

*Halomonas boliviensis* is a gammaproteobacterium that is known to assimilate and convert various carbohydrates into PHB [15]. Owing to this capability, it has been used to synthesize the polyester using hydrolysates of starch [16] or wheat bran [17]. The PHB accumulation in the cells was between 30-56 % (wt.) induced by the limitation of yeast extract that was used as the nitrogen source in the culture medium [16, 17]. Furthermore, *H. boliviensis* also produces large amounts of PHB when the synthesis of the polymer is triggered by the depletion of monosodium glutamate (MSG), which was used as a much cheaper nitrogen source than yeast extract [5]. Studies on the genome sequence of the bacterium suggest that maltose is first assimilated by action of an α-glucosidase, before it is metabolized via the glycolytic pathway [18]. On the other hand, the assimilation of glucose by *H. boliviensis* has not been described yet. Both glucose and maltose can be encountered in starch hydrolysates of agricultural residues [16], which are regarded as inexpensive and suitable substrates for PHA production. To understand the metabolic routes that allow the assimilation of such carbohydrates by a microorganism for polyester production is hence important.

This research article describes the production of PHB by *H. boliviensis* using combinations of glucose, xylose and maltose as carbon sources, and MSG as the nitrogen limiting nutrient in the culture medium. *Halomonas boliviensis* was also grown in an ALR containing starch hydrolysate. The hydrolysate was employed as the main carbon source for production of PHB. Moreover, the assimilation of glucose in the Entner-Doudoroff and the pentose phosphate pathways was deduced from an evolutionary analysis of the genome of this bacterium.

## Results

### Production of PHB by *H. boliviensis* using different carbohydrates

This set of experiments was performed in shake flasks containing PHB production medium at 35 °C and 200 rpm for 30 hrs. MSG was used as the nitrogen limiting source at a concentration of 0.2 % (w/v) as determined previously [18]. As shown in Table 1, mixtures of carbohydrates such as those obtained from partially hydrolyzed starch [16] and wheat bran [17], the starch hydrolysate used in this study, and a combination of the starch hydrolysate and xylose were used as carbon sources for PHB production. The highest CDW was reached when starch hydrolysate was utilized as carbon source, whilst the lowest CDW was attained when a combination of both glucose and maltose was included in the culture medium (Table 1). The mixtures of glucose and xylose or starch hydrolysate and xylose led to similar CDW values (Table 1). Moreover, the highest PHB yields, between 56 and 61 % (wt.) (Table 1), were achieved when either starch hydrolysate or the mixture of glucose and xylose were used as carbon sources. Furthermore, the high Residual Cell Mass (RCM) and PHB volumetric productivity obtained when the starch hydrolysate was used as a source for PHB production suggest that it also favors both cell growth and PHB synthesis (Table 1).

**Table 1:** Cell growth and PHB production attained by *H. boliviensis* in shake flasks using different carbon sources. Samples were withdrawn after 30 h of cultivation. The experiments were performed in triplicate at a temperature of 35 °C and an agitation speed of 200 rpm; standard deviations of the mean values are shown in parentheses. Abbreviations refer to cell dry weight (CDW) and residual cell mass (RCM)

Carbon source	CDW	PHB	Volumetric productivity	RCM
	(g/L)	wt.%	g/L/h	(g/L)
Glucose and xylose	8.1 (±0.2)	61 (±2.7)	0.15	3.2
Glucose and maltose (weight ratio of 0.7:0.3)	7.0 (±0.1)	53 (±3.0)	0.12	3.3
Starch hydrolysate and xylose	8.3 (±0.1)	45 (±1.1)	0.12	4.6
Starch hydrolysate	9.2 (±0.3)	56 (±2.6)	0.16	4.1

### PHB production by *H. boliviensis* in an ALR

**Fig. 1:**
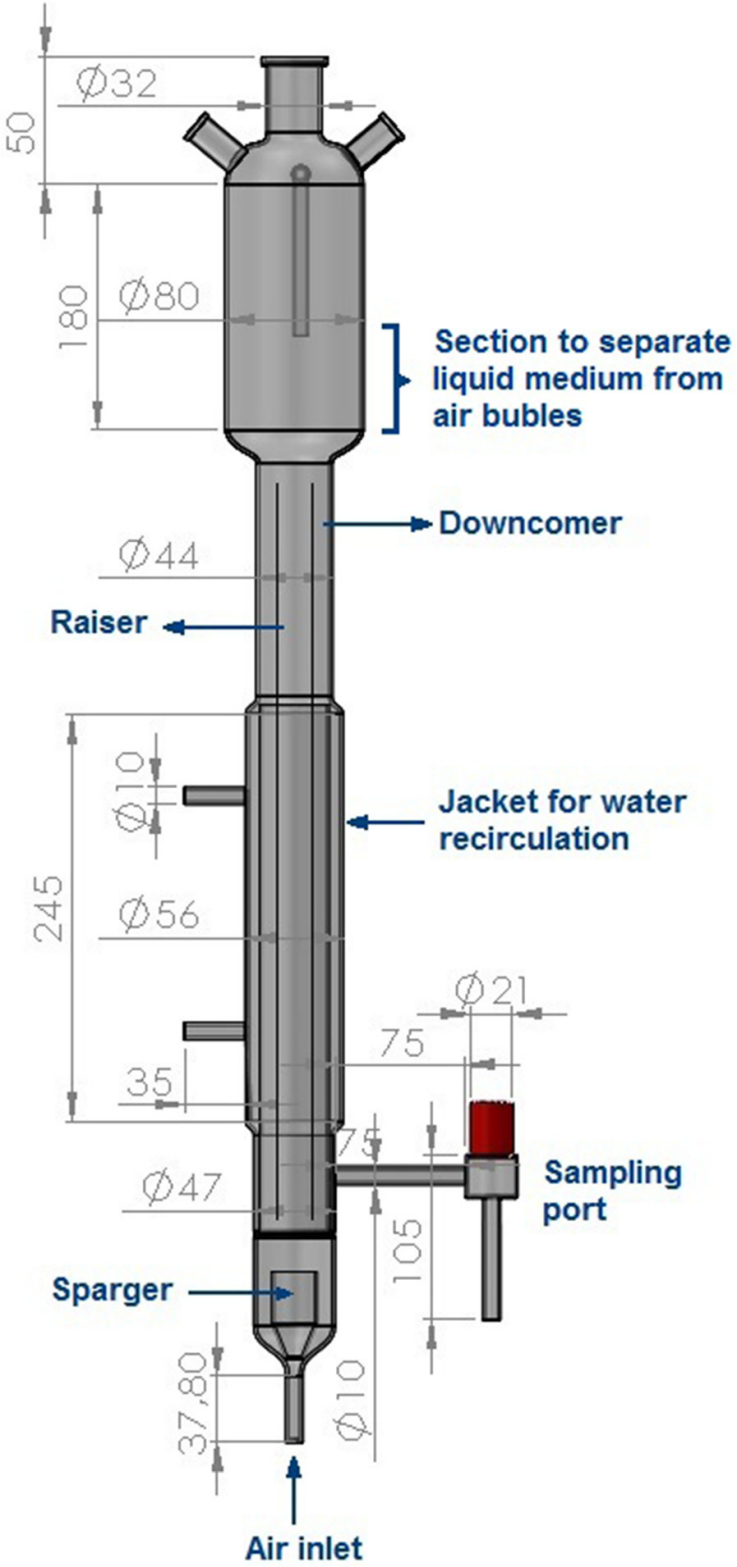
Glass ALR used for the production of PHB by *H. boliviensis*. All dimensions are given in mm. Different sections of the reactor are also depicted

**Fig. 2:**
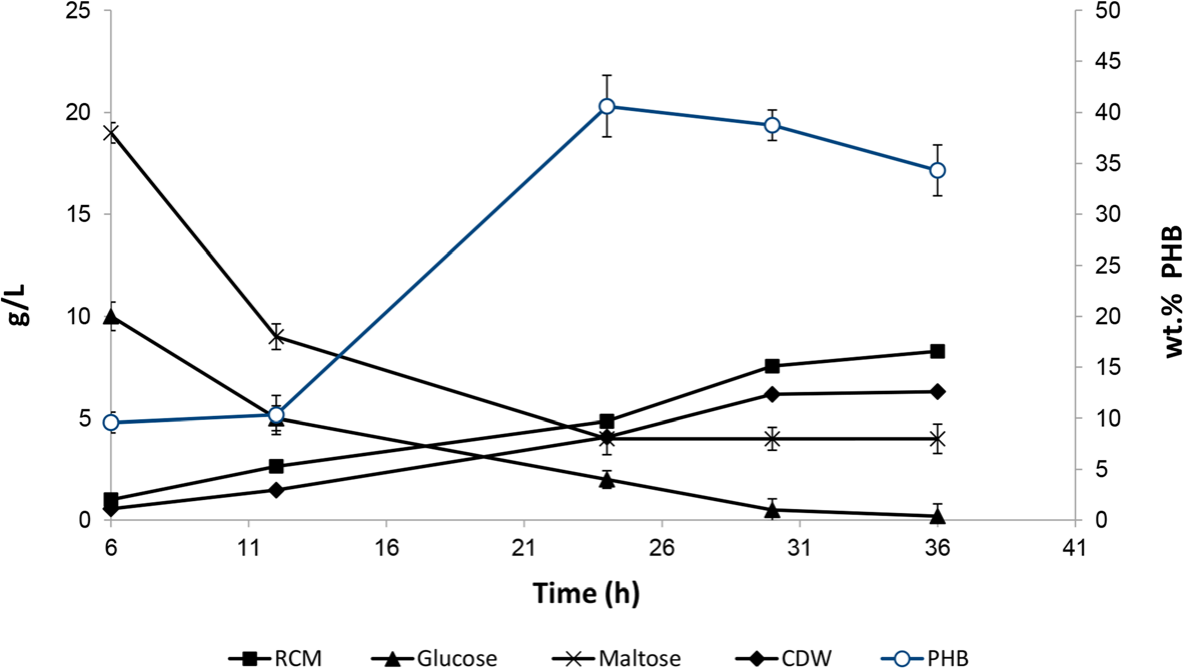
Cell growth, PHB production, and carbohydrate uptake by *H. boliviensis* as a function of production time in an ALR operated in a batch system. Air flow rate into the reactor was set at 2.0 L min^−1^, pH was maintained at 7.5 ± 0.3 and water at 35 °C was circulated by the jacket of the reactor. The initial MSG concentration was 2 g L^−1^. The experiments were performed in triplicate

Starch hydrolysate was used as carbon source in an ALR (Fig. 1). CDW, PHB accumulation, RCM, and glucose and maltose consumed by the bacterium as a function of production time are shown in Fig. 2. Glucose and maltose in the starch hydrolysate were rapidly assimilated and used mainly in the formation of cell biomass up to the first 12 hrs of cultivation; during this time period, *H. boliviensis* yielded only 10.5 % (wt.) PHB (Fig. 2), with maltose being assimilated faster than glucose. Between 12 and 24 hrs of cultivation, glucose and maltose were consumed at almost the same rate, with concomitant increase in PHB production that reached a maximum of 41 % (wt.) (Fig. 2). The onset of PHB depolymerization coincided with low concentrations of glucose and maltose in the medium. During this time, cells continued to grow as observed by the increase in RCM, although they were only using glucose as carbon source.

### Assimilation of carbohydrates by *H. boliviensis* by the Entner-Doudoroff and pentose phosphate pathways

**Fig. 3:**
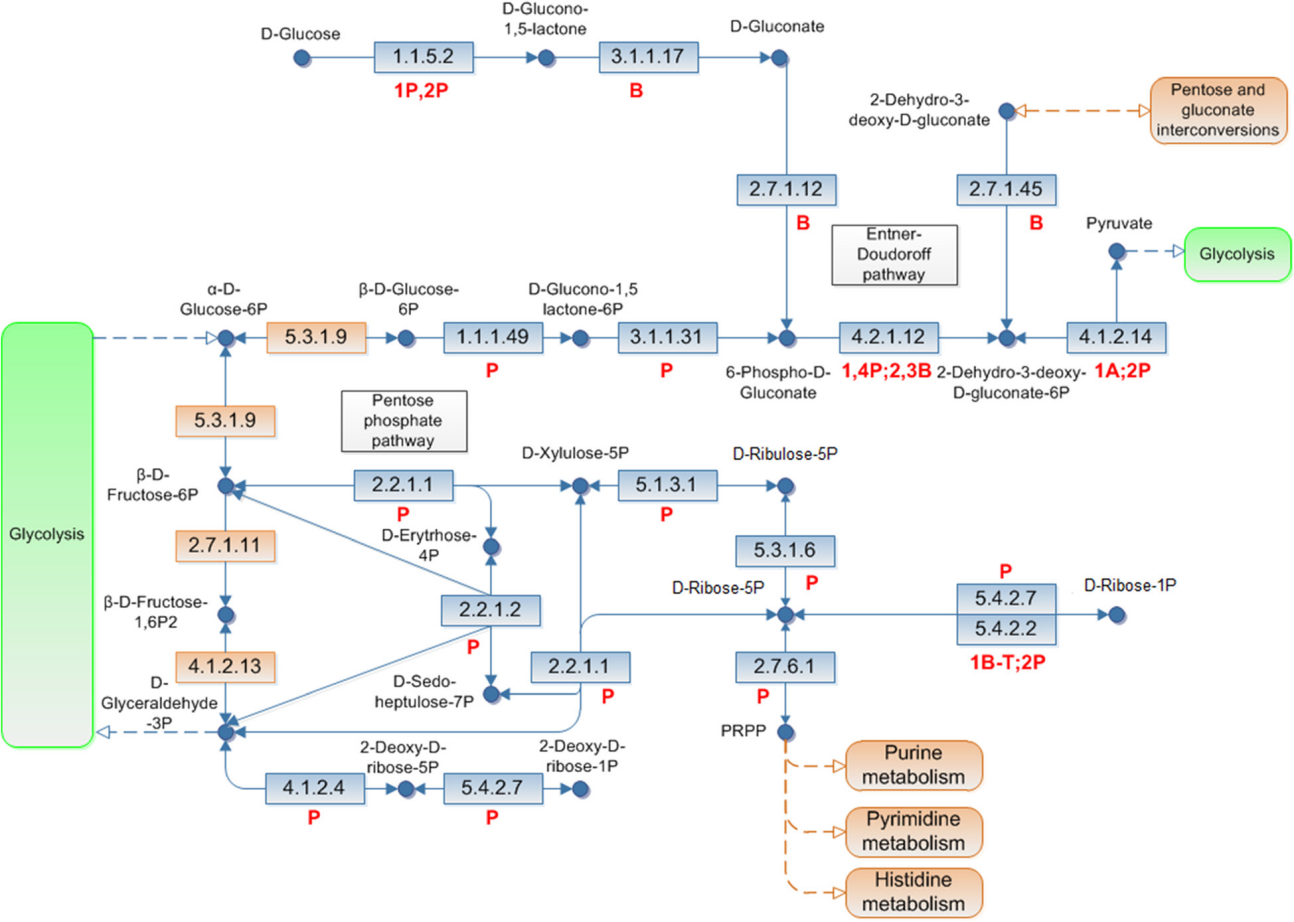
The Entner-Doudoroff and the pentose phosphate pathways in *H. boliviensis*. Enzymes highlighted in blue were the subject of this study. Numbers and abbreviations for each metabolic step refer to the number of alleles and the cluster that the alleles formed with: P, Proteobacteria; B, bacteria; T, thermophilic archaea; A, non-thermophilic archaea and combinations of these groups of organisms. Database accession numbers for enzymes of *H. boliviensis* are provided in Table 2. EC numbers for the enzymes in the metabolisms are pointed out as classified in the KEGG pathway database, and are listed in Table 2

The rate of consumption of glucose and maltose in the starch hydrolysate by *H. boliviensis* had distinctive patterns during both cell growth and polymer production (Fig. 2). The genome sequence of *H. boliviensis* was studied in order to determine the biochemical pathways in which these carbohydrates are assimilated. Enzymes involved in the Entner-Doudoroff (E-D) and pentose phosphate (PP) pathways were found to be involved (Fig. 3). Glucose is metabolized through the E-D pathway which initially involves two alleles of putative quinoprotein glucose dehydrogenases (E.C. 1.1.5.2), (Fig. 3, Table 2). A phylogenetic analysis of the protein sequences of these alleles revealed that they have diverged among enzymes of other Proteobacteria (Additional file 1: Figure S1). This phylogenetic cluster also included two alleles found in *Halomonas* sp. TD01 (Additional file 1: Figure S1); however the alleles in *H. boliviensis* did not share an evolutionary relationship with enzymes of *H. elongata* and *C. salexigens* (Additional file 1: Figure S1, Table 2). The E-D pathway in *H. boliviensis* continues with a gluconolactonase (E.C. 3.1.1.17) (Fig. 3) that formed a phylogenetic group with gluconolactonases belonging to bacteria not included among the Proteobacteria (Additional file 2: Figure S2, Table 2). Similar evolutionary analyses were performed on the remaining enzymes of the E-D pathway. Some of these enzymes have evolved along with enzymes of bacteria non-taxonomically related to *H. boliviensis*, and some enzymes have even diverged together with enzymes of archaea (Fig. 3).

**Table 2:** Enzymes present in the pentose phosphate pathway of strains of the family *Halomonadaceae*. Enzymes and their alleles in *H. boliviensis* were taken as reference; public data base accession numbers for these enzymes are also shown in the table. Correspondingly enzymes and alleles were sought in *Halomonas* sp. TD01, *H. elongata* and *C. salexigens*. Symbols refer to: +, the enzyme is present in a phylogenetic cluster with *H. boliviensis* and −, the enzyme is absent in a phylogenetic cluster with *H. boliviensis*

Enzyme	E.C. number	Accession number	COG	Alleles in *H. boliviensis*	*Halomonas sp.* TD01	*H. elongata*	*C. salexigens*
Quinoprotein glucose dehydrogenase	1.1.5.2	WP_007112539	4993	1	+	-	-
		WP_007111394		2	+	-	-
Gluconolactonase	3.1.1.17	WP_007113046	3386	1	-	-	-
Gluconokinase	2.7.1.12	WP_007111165	3265	1	+	-	+
Phosphogluconate dehydratase	4.2.1.12	WP_007112813	0129	1	-	+	+
		WP_007114498		2	+	+	-
		WP_007113381		3	+	+	+
		WP_007113033		4	+	+	+
2-dehydro-3-deoxygluconokinase	2.7.1.45	WP_007112577	0524	1	+	+	-
		WP_007114584		2	-	+	+
		WP_007113036		3	+	+	+
2-dehydro-3- deoxyphosphogluconate aldolase	4.1.2.14	WP_007113045	0800	1	-	-	-
		WP_007113048		2	+	+	+
6-phosphogluconolactonase	3.1.1.31	WP_007113049	0363	1	+	+	+
Glucose-6-phosphate 1-dehydrogenase	1.1.1.49	WP_007113050	0364	1	+	+	+
Transketolase	2.2.1.1	WP_007112132	0021	1	+	+	+
Transaldolase	2.2.1.2	WP_007111145	0176	1	-	+	+
Ribulose-phosphate 3-epimerase	5.1.3.1	WP_007113254	0036	1	+	+	+
Ribose 5-phosphate isomerase	5.3.1.6	WP_007112167	0120	1	+	+	+
Deoxyribose-phosphate aldolase	4.1.2.4	WP_007111255	0274	1	+	+	+
Phosphopentomutase	5.4.2.7	WP_007111257	1015	1	+	+	-
Ribose-phosphate pyrophosphokinase	2.7.6.1	WP_007113450	0462	1	+	+	+
Phosphoglucomutase	5.4.2.2	WP_007111739	1109	1	+	+	+
		WP_007111831		2	-	+	+

*Halomonas boliviensis* may take up glucose by a phosphotransferase – phosphoenol pyruvate – system which phosphorylates the sugar into D-glucose-6-phosphate (G6P) (Enzyme database accession number: WP_007113062) and/or it may transport glucose by an ABC multisugar transport system (Enzymes database accession numbers: WP_007113038, WP_040480617, WP_007113040, WP_007113041) (data not shown). Moreover, it was previously reported that other carbohydrates such as maltose and sucrose should yield G6P in *H. boliviensis* [18]. G6P can continue through the E-D pathway by the action of a glucose-6-phosphate-1-deshydrogenase (E.C. 1.1.1.49) and a 6-phosphogluconolactonase (E.C. 3.1.1.31) (Fig. 3). G6P should also initiate the glycolysis and the pentose phosphate (PP) pathway (Fig. 3). The PP pathway results in the synthesis of two important intermediates: ribose-5-phosphate (R5P), used in the synthesis of nucleotides and nucleic acids, and erytrose-4-phosphate (E4P), used in the synthesis of aromatic amino acids. Importantly, we did not find genes related to the expression of 6-phosphogluconate dehydrogenase (E.C. 1.1.1.43); this enzyme allows the conversion of 6-phospho-D-gluconate into R5P together with the generation of a molecule of NADPH, whereby D-glucose assimilation cannot be transformed to R5P neither via the E-D pathway nor via the PP pathway (Fig. 3). Furthermore, our evolutionary studies showed that all enzymes of *H. boliviensis* in the PP pathway have evolved within phylogenetic clusters along with enzymes of other Proteobacteria (Fig. 3), and most of these enzymes were included in the same cluster with enzymes found in *Halomonas* sp. TD01 and *Halomonas elongata* (Table 2).

## Discussion

*Halomonas boliviensis* is a halophilic bacterium that is known to produce large amounts of PHB when cultured using carbon sources such as glucose, sucrose, butyric acid, acetate and others [15]. Nevertheless, cheap carbon sources such as hydrolysates of agroindustrial residues are usually composed by a combination of carbohydrates [16, 17]. Therefore a combination of different sugars and starch hydrolysate were used in our experiments (Table 1). We hydrolyzed cassava starch using *Fungamyl* – an α-amylase that is commonly utilized in industrial processes to partially hydrolyse starch. We also considered that complete hydrolysis of starch into glucose was not necessary, since *H. boliviensis* is able to assimilate both glucose and maltose [16]. Hence, controlled hydrolysis was carried out to obtain a hydrolysate containing glucose and maltose in a weight ratio of 1:2. We also used sodium glutamate as a cheap nitrogen source that limits cell growth after its depletion from the culture medium. Starch hydrolysate induced both cell growth and PHB accumulation (Table 1), whereas the combination of glucose and maltose at a weight ratio of 0.7:0.3 decreased, to some extent, cell biomass and PHB yield (Table 1). Reports show that *H. boliviensis* is able to attain 9 g L^−1^ CDW and 55 % (wt.) PHB when yeast extract is used as nitrogen source, whilst 30-56 % (wt.) PHB is found in the cells when starch or wheat hydrolysates are used as the carbon source [16, 17]. The report also indicates that use of maltose as sole carbon source by the microorganism enable it to accumulate of 58.8 % (wt.) PHB [16]. Amounts of PHB produced in this study and those accomplished when using yeast extract is used as nitrogen source are similar. Therefore, partially hydrolyzed starch and MSG can be used as cheap carbon and nitrogen sources for PHB synthesis by *H. boliviensis* (Table 1).

A simple and efficient PHB production system is as important as the availability of cheap carbon and nitrogen sources. Therefore we designed and built an ALR with a simple configuration (Fig. 1). Cells were then cultured in the reactor for PHB production using starch hydrolysate and MSG. Temperature, pH and air flow into the reactor were controlled during the process. The maximum PHB content reached in our experiments, i.e. 41 % (wt.) (Fig. 2), was similar to that reported for *B. sacchari* [13], but was lower than those found in *C. necator* [12] and *A. australica* [14], ~ 70-73 % (wt.). In all cases, the microorganisms were cultivated in ALR. The maximum CDW attained by *H. boliviensis* was 12.3 g L^−1^, similar to that reported for *A. australica* (10.8 g L^−1^) [14]. *Burkholderia sacchari* reached a very high cell density (150 g L^−1^), although step-wise addition of sucrose solution into the reactor was involved [13]. In fed-batch fermentations using STRs, *H. boliviensis* has been reported to attain higher cell concentrations and PHB contents than in the ALR [5]. An excess of a carbon source in the culture medium of a PHB producing microorganism is a prerequisite for optimum production of the polymer, therefore glucose or an appropriate ratio of glucose and maltose (Fig. 2) fed to the ALR during the PHB production phase may improve cell growth and PHB yield.

An increase in the value of RCM during cell growth of *H. boliviensis* suggests that the cells were producing PHB while they were still growing. *Azohydromonas australica* grows, while it synthesizes PHB [14]; however, this is rarely observed in other microorganisms [1]. The growth of *H. boliviensis* was not as fast as that observed in *A. australica*, but the former commonly produces PHB while the RCM is constant [15]. Indeed, *H. boliviensis* did not grow during the production of PHB when a sole carbon source such as glucose or butyric acid was included in its culture medium [15], albeit the RCM showed a similar trend to that found in Fig. 1, when a mixture of sucrose and glucose was used in the production process [18]. The intracellular concentrations of CoA, NADPH and NADH have been shown to play crucial roles in both cell growth and PHB synthesis [19]. For example, high concentrations of CoA, usually found during active growth of *Methylobacterium rhodesianum*, strongly inhibited PHB synthesis, whereas cell growth was hindered by large amounts of NADH due to its inhibitive effect on citrate synthase; furthermore, the NADPH concentration had to be sufficiently high to prompt the synthesis of the polyester [19]. *Methylobacterium rhodesianum* produces PHB during its stationary phase of growth when the cells are grown on methanol [20]. However, when cultures of *M. rhodesianum* were first grown on methanol and then shifted to fructose, they were found to produce PHB during the cell growth [20]. In these experiments, the intracellular concentration of CoA decreased from a high value during the cell growth on methanol to a low value after the shift to fructose [20]. Different carbon sources may be metabolized through distinct pathways, which may lead to different intracellular concentrations of the reducing energy equivalents [NAD(P)H] and CoA. The combination of carbohydrates used as carbon sources for the growth of *H. boliviensis* (Fig. 2) appears to balance the intracellular energy and CoA concentrations so that cell growth and PHB synthesis proceed simultaneously.

The evolutionary trends of all enzymes involved in the E-D and PP pathways in *H. boliviensis* revealed that most enzymes evolved in phylogenetic clusters formed by proteins from other halomonads (Table 2) and Proteobacteria (Fig. 3). *Halomonas boliviensis* is a gammaproteobacterium, hence its enzymes should be phylogentically related to other enzymes found in Proteobacteria. This however is not the case with gluconolactonase and a gluconokinase at the third and second steps of the PP pathway in *H. boliviensis*. These enzymes appear to have evolved in groups from enzymes belonging to non-taxonomically related bacteria (e.g., Additional file 2: Figure S2), implying that they, together with those that clustered with archaeal enzymes, were acquired by horizontal gene transfer (HGT) [18, 21]. HGT was also found in the glycolytic pathway of *H. boliviensis* [18]. The fourth step in the PP pathway is catalyzed by a 6-phosphogluconate dehydrogenase (E.C. 1.1.1.43), which is absent in *H. boliviensis*. This enzyme has proven to play an important role in bacterial cell growth; for instance, mutants of *Gluconobacter oxydans* [22] and *Pseudomonas cepacia* [23] deficient in 6-phosphogluconate dehydrogenase showed growth inhibition when mannitol and glucose were used as carbon sources, respectively. *Halomonas boliviensis* seems to have overcome the lack of this enzyme by favoring a rapid assimilation of maltose (Fig. 2) via the glycolysis, leading to the generation of precursors of nucleotides, nucleic acids and aromatic amino acids in the PP pathway. A molecule of glucose yields 1 ATP, 1 NADH and 1 NADPH in the E-D pathway. By comparison, glycolysis has a net yield of 2 ATP and 2 NADH. Assimilation of glucose and maltose in both of these cellular metabolic routes appears to equilibrate the intracellular energy for PHB production and anabolism (Fig. 2). Moreover, PHB depolymerization, which commonly involves hydrolysis of ATP, requires a less amount of energy than an anabolic metabolism, and only demanded the assimilation of glucose by *H. boliviensis* (Fig. 2); this sugar may have been assimilated by the E-D pathway because precursors for DNA and amino acids were not as essential as they were during the first 24 hrs of the cultivation during the active cell growth. Further studies are being performed on PHB production in an air-lift reactor that is fed with an automated inflow of carbons sources.

## Conclusion

This study showed that the production of PHB by *H. boliviensis* using cheap substrates such as starch hydrolysate in a simple production system involving an ALR is feasible. Both maltose and glucose in the hydrolysate induce cell growth and PHB synthesis; most likely the cells balance adequately CoA and NAD(P)H during the assimilation of these carbohydrates. The combination of cheap substrates, simple production systems and the use of non-strict sterile conditions by the halophile *H. boliviensis* are desirable traits for large scale production of PHB, and should lead to a competitive bioprocess. However, additional studies on air-lift reactors operated under a fed-batch mode are required.

## Methods

### Bacterial strain and maintenance

*Halomonas boliviensis* LC1^T^ (= DSM 15516^T^) was maintained at 4 °C on solid HM medium [15], containing (%, w/v): NaCl, 4.5; MgSO_4_ · 7H_2_O, 0.025; CaCl_2_ · 2H_2_O, 0.009; KCl, 0.05; NaBr, 0.006; peptone, 0.5; yeast extract, 1.0; glucose, 0.1; and agar, 2.0. The pH of the medium was adjusted to 7.5 using 3 M NaOH.

### Hydrolysis of starch obtained from cassava

Starch from cassava was suspended in 0.05 M citrate buffer solution (pH = 6) in a stirred tank, after which α-amylase, *Fungamyl*, was added. Hydrolysis of starch was allowed to proceed at 60 °C and at a stirring speed of 200 rpm for 24 hrs. The total sugar concentration in the resulting hydrolysate solution was determined to be 200 g L^−1^ containing mainly glucose and maltose in a weight ratio of 1:2.

### Culture medium and conditions for PHB production in shake flasks

Seed culture and PHB production media were formulated as described previously [16]. Seed culture medium contained (%, w/v): NaCl, 2.5; MgSO_4_°7H_2_O, 0.25; K_2_HPO_4_, 0.05; NH_4_Cl, 0.23; FeSO_4_°7H_2_O, 0.005; glucose, 1.0; monosodium glutamate (MSG), 0.2 and TRIS, 1.5. The seed culture was at 30 °C and 200 rpm for 15 hrs (OD_600_ = 0.5-0.55; CDW = 1.0 ± 0.1 g L^−1^). 5 % (v/v) was used to inoculate the PHB production medium, containing (%, w/v): NaCl, 2.5; MgSO_4_°7H_2_O, 0.5; K_2_HPO_4_, 0.22; NH_4_Cl, 0.4; FeSO_4_°7H_2_O, 0.005; MSG, 0.2; and the following carbohydrates (%, w/v): 1) 1.5 glucose and 0.5 xylose, 2) 1.4 glucose and 0.6 maltose, 3) 1.5 starch hydrolysate and 0.5 xylose, 4) 2.5 starch hydrolysate, respectively. A low amount, 0.2 % (w/v) MSG, was added to the production medium to induce its depletion by *H. boliviensis* during the cultivation. All experiments were performed in 1000 ml shake flasks at 35 °C and 200 rpm of agitation.

### Air-lift reactor construction and operation for PHB production

An air-lift glass with geometrical dimensions as depicted in Fig. 1 was used in this study. Air at a flow rate of 2 L min^−1^ (2.5 vvm) was pumped through a millipore glass net that acted as air sparger. Air bubbles created by the sparger enter to an inner tube (known as the riser) which is concentrically surrounded by an ungassed inner tube (known as the downcomer). The different gas holdup in the raiser and downcomer zones result in dissimilar bulk densities of the liquid culture medium in these regions which causes circulation of the liquid in the reactor by gas-lift action. On top of the riser and downcomer a column section of 180 mm height was placed in order to separate air bubbles from the culture medium. The upper most section of the reactor had three openings: the middle one for inserting a pH electrode and the ones on either side as an air outlet and for addition of acid, base or antifoam to the reactor, respectively. Moreover on one side of the downcomer, and at 105 mm from the sparger, a sampling device was coupled. A 245 mm height external jacket was also included in the reactor to maintain the temperature constant by water recirculation.

*Halomonas boliviensis* was cultured in a 1 L Erlenmeyer flask containing 50 ml of seed culture medium at 30 °C for 15 hrs. The seed culture was inoculated in 700 ml of PHB production medium in the reactor and cultivation was allowed to proceed for 36 hrs. pH of the medium was controlled at 7.5 ± 0.3 by adding 3 M NaOH or 3 M HCl in the reactor whenever a deviation from the set point value was observed. Water at 35 °C originating from a thermostatic water bath was recirculated through the jacket of the reactor. Antifoam was added when needed. Samples were withdrawn from the reactor at different time intervals.

### Genome sequences studied and evolutionary analysis of proteins

Four genome sequences corresponding to strains of the family *Halomonadaceae* were selected for this study. The strains were *Halomonas boliviensis* LC1^T^ (=DSM 15516^T^) [18], *Halomonas elongata* DSM 2581^T^ [24], *Halomonas* sp. TD01 [25], and *Chromohalobacter salexigens* DSM 3043 [26].

A total of 6901 alignments of clusters of orthologous proteins (COGs) of 88 microorganisms, as classified in COGs [27] and EggNOG [28] databases, were obtained from that described by Puigbò *et al*. [29]. The protein sequences of these 88 microorganisms were used as reference for the evolutionary analysis. Protein sequences of strains of the family *Halomonadaceae* were included in the alignments with the references for each corresponding COG using the Muscle program [30] included in the MEGA 5 software package [31] with default parameters. Unrooted maximum likelihood phylogenetic trees for each COG were constructed using MEGA 5 under a WAG with frequencies (+F) model, with uniform mutation rates among amino acid sites and complete deletion of gaps and missing data.

### Determination of biochemical pathways

*Halomonas boliviensis* was taken as the reference strain for the determination of biochemical pathways. The functional annotation of enzymes involved in the pentose phosphate and Entner-Doudoroff pathways was accomplished by analyses of protein sequences. Genes of *H. boliviensis* were aligned to others in databases to attain its corresponding functional annotation. To ensure the biological meaning, only one high-quality information as annotation to the genes from many results was chosen. BLAST was used to accomplish functional annotation combined with different databases. BLAST version: blastall 2.2.21 software (provided by the National Center for Biotechnology Information) was used for these studies. Alignment results were obtained using the KEGG, COG, SwissProt, TrEMBL, and NR databases. A BLAST search was also used to find enzymes in *H. elongata*, *C. salexigens*, and *Halomonas* sp. TD01 that share high identities with those annotated for *H. boliviensis.*

### Quantitative analyses

Cell dry weight (CDW) and PHB content in *H. boliviensis* were determined as reported previously [15]. Residual cell mass (RCM) concentration was calculated as the difference between the CDW and PHB concentration, while PHB content (wt.%) was obtained as the percentage of the ratio of PHB concentration to the CDW as defined by Lee *et al*. [32]. All analyses were performed in triplicate. Glucose and maltose were determined using the same HPLC system with a Polypore CA column (Perkin-Elmer), a RI detector at 80 °C and water as mobile phase at a flow rate of 0.3 ml min^−1^.

## Additional files

Additional file 1: Figure S1. Phylogentic tree relating clusters of orthologous protein sequences (COGs) corresponding to quinoprotein glucose dehydrogenase COG4993. The tree was constructed under a maximum likelihood approach using MEGA 5 software and the WAG with frequencies (+F) model. GenBank accession numbers of the sequences are given in parentheses. Numbers at branch points are bootstrap values (500 replicates). Bar denotes 0.2 sequence divergence. (PDF 60 kb) Additional file 2: Figure S2. Phylogentic tree relating clusters of orthologous protein sequences (COGs) corresponding to gluconolactonase COG3386. The tree was constructed under a maximum likelihood approach using MEGA 5 software and the WAG with frequencies (+F) model. GenBank accession numbers of the sequences are given in parentheses. Numbers at branch points are bootstrap values (500 replicates). Bar denotes 0.2 sequence divergence. (PDF 75 kb)
