# Supplementary figures and images for: Production of poly(3-hydroxybutyrate) by Halomonas boliviensis in an air-lift reactor
Source: J Biol Res (Thessalon). 2015 Aug 3;22(1):8. doi: 10.1186/s40709-015-0031-6 (PMC4522284; doi:10.1186/s40709-015-0031-6)

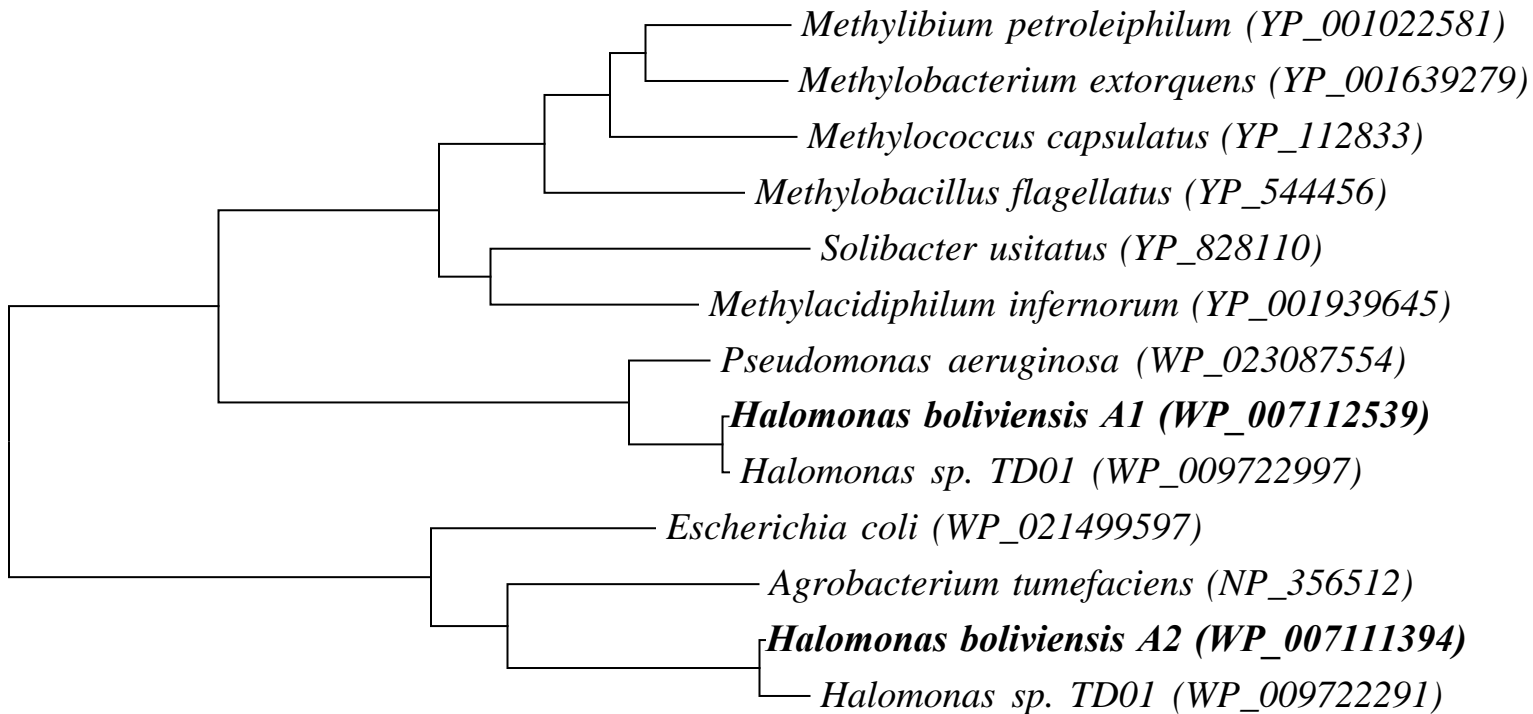

0.2

Supplement: Additional file 1: Figure S1. — Phylogentic tree relating clusters of orthologous protein sequences (COGs) corresponding to quinoprotein glucose dehydrogenase COG4993. The tree was constructed under a maximum likelihood approach using MEGA 5 software and the WAG with frequencies (+F) model. GenBank accession numbers of the sequences are given in parentheses. Numbers at branch points are bootstrap values (500 replicates). Bar denotes 0.2 sequence divergence. (PDF 60 kb) [file 40709_2015_31_MOESM1_ESM.pdf]

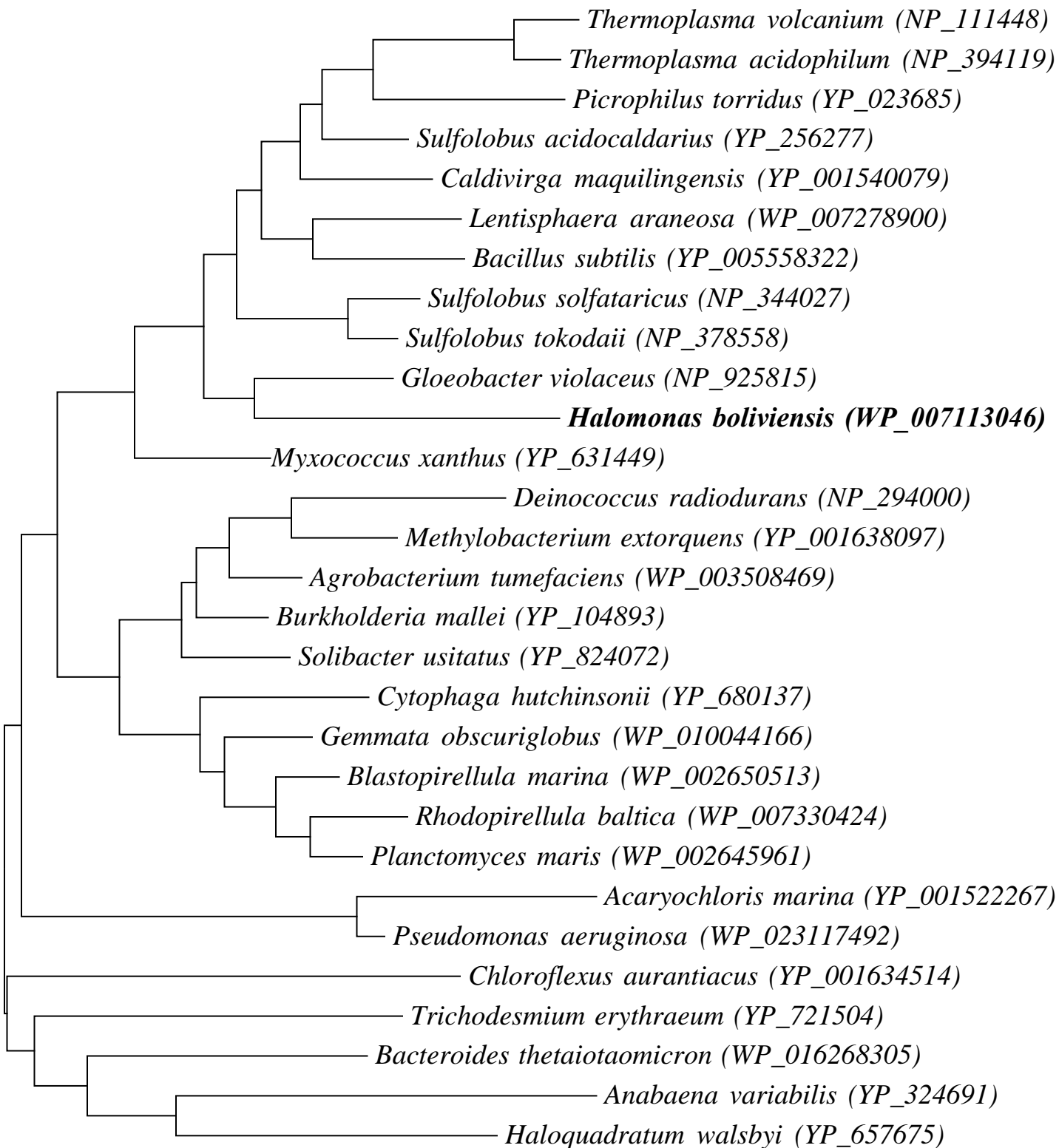

0.2

Supplement: Additional file 2: Figure S2. — Phylogentic tree relating clusters of orthologous protein sequences (COGs) corresponding to gluconolactonase COG3386. The tree was constructed under a maximum likelihood approach using MEGA 5 software and the WAG with frequencies (+F) model. GenBank accession numbers of the sequences are given in parentheses. Numbers at branch points are bootstrap values (500 replicates). Bar denotes 0.2 sequence divergence. (PDF 75 kb) [file 40709_2015_31_MOESM2_ESM.pdf]
